# Captive-reared migratory monarch butterflies show natural orientation when released in the wild

**DOI:** 10.1093/conphys/coab032

**Published:** 2021-05-11

**Authors:** Alana A E Wilcox, Amy E M Newman, Nigel E Raine, Greg W Mitchell, D Ryan Norris

**Affiliations:** 1 Department of Integrative Biology, University of Guelph, Guelph, Ontario, N1G 2W1, Canada; 2 School of Environmental Sciences, University of Guelph, Guelph, Ontario, N1G 2W1, Canada; 3 Wildlife Research Division, Environment and Climate Change Canada, National Wildlife Research Centre, 1125 Colonel By Drive, Ottawa, Ontario, K1S 5B6, Canada; 4 Department of Biology, Carleton University, 1125 Colonel By Drive, Ottawa, Ontario, K1S 5B6, Canada; 5 Nature Conservancy of Canada, 245 Eglington Avenue East, Toronto, Ontario, M4P 3J1, Canada

**Keywords:** *Danaus plexippus*, insect migration, pollinator conservation, radio tracking

## Abstract

Eastern North American migratory monarch butterflies (*Danaus plexippus*) have faced sharp declines over the past two decades. Captive rearing of monarch butterflies is a popular and widely used approach for both public education and conservation. However, recent evidence suggests that captive-reared monarchs may lose their capacity to orient southward during fall migration to their Mexican overwintering sites, raising questions about the value and ethics of this activity undertaken by tens of thousands of North American citizens, educators, volunteers and conservationists each year. We raised offspring of wild-caught monarchs on swamp milkweed (*Asclepias incarnata*) indoors at 29°C during the day and 23°C at night (~77% RH, 18L:6D), and after eclosion, individuals were either tested in a flight simulator or radio tracked in the wild using an array of automated telemetry towers. While 26% (10/39) of monarchs tested in the flight simulator showed a weakly concentrated southward orientation, 97% (28/29) of the radio-tracked individuals that could be reliably detected by automated towers flew in a south to southeast direction from the release site and were detected at distances of up to 200 km away. Our results suggest that, although captive rearing of monarch butterflies may cause temporary disorientation, proper orientation is likely established after exposure to natural skylight cues.

## Teaser

Recent work suggests captive-reared monarch butterflies lose their capacity to fly southward in fall, questioning the value of captive rearing. We show that, in contrast to captive-reared monarchs flown in a flight simulator, monarchs reared under laboratory conditions and released in the wild showed proper southward orientation.

## Introduction

Captive rearing and the reintroduction of animals into the wild has been an effective tool for mitigating the decline of some species at risk (Hughes and Bennett, 1991). Capacity for acclimation in captivity varies among species (Chamove *et al.,* 1988; Mettke, 1995; Mason, 2010), with some species being notoriously difficult to maintain or having lower reproductive success in captivity (Bauman *et al.,* 2010; Mason, 2010). Behaviour is known to differ between captive and wild populations of mammals (Blanchard *et al.,* 1986), fish (Gro Vea Salvanes and Braithwaite, 2006) and insects (Ings *et al*., 2009; Fisher *et al.,* 2015), with well-documented incidents of abnormal behaviour in captive mammal populations (McPhee, 2004; Birkett and Newton-Fisher, 2011).

Captive rearing of monarch butterflies (*Danaus plexippus*) is an extremely popular activity across North America, but recent scientific evidence calls into question the utility and ethics of captive rearing in this species. In late fall, monarch butterflies migrate up to 4000 km from the mid-western and north-eastern United States and south-eastern Canada to Mexico (Urquhart, 1960; Urquhart and Urquhart, 1978; Brower, 1995). Some studies have suggested that monarchs in eastern North America have experienced severe declines over the past two decades (Brower *et al*., 2012; Thogmartin *et al.*, 2017), with evidence, in part, suggesting that this may be linked to the widespread loss of their host plant milkweed (*Asclepias* spp.; Pleasants and Oberhauser, 2012; Flockhart *et al*., 2012; Pleasants, 2016). Each year, tens of thousands of educators, citizens, volunteers and conservationists engage in efforts to rear monarchs to adulthood (while minimizing risks, see Monarch Joint Venture, 2018), monitor their abundance and movements (Howard and Davis, 2004; Oberhauser and Prysby, 2008; Ries and Oberhauser, 2015) and educate the public about the biology and natural history of butterflies (Kountoupes and Oberhauser, 2008). However, recent studies have suggested that there is potential for long-term behavioural changes of captive-reared monarchs intended to be released in the wild during the fall migratory period (Tenger-Trolander *et al.,* 2019; Tenger-Trolander and Kronfrost, 2020).

Recent studies have shown that, when raised indoors or in chambers [i.e. reared from eggs indoors in autumn-like conditions until adult emergence (eclosion)], monarch butterflies do not show normal southern orientation (Tenger-Trolander **et al.*,* 2019), even when exposed to sunlight through a window during development (Tenger-Trolander and Kronfrost, 2020). These results were obtained when individual adult butterflies were tested in a confined flight simulator that measured directional orientation. The authors concluded that the activity of rearing captive monarchs for release would be an ineffective conservation practice to help boost migratory populations. These results also question the general ethics of monarch rearing that is done each year by hobbyists and educators across North America. However, the possibility remains that monarch butterflies released in the wild are able to show proper orientation if they can calibrate their internal compass with exposure to natural skylight that provides external cues critical to the functioning of the molecular clock that governs directional flight (Reppert *et al.,* 2010).

In this study, we sought to understand whether captive-reared monarch butterflies could show proper orientation in a natural southward direction during fall migration when released in the wild. To do this, we reared monarch butterflies in captivity and then, similar to previous studies (Tenger-Trolander **et al.*,* 2019; Tenger-Trolander and Kronfrost, 2020), tested them in a confined flight simulator. However, we also tested another group of monarch butterflies raised under the same captive conditions, but released in the wild and then subsequently radio tracked using an array of over 100 automated telemetry towers (Motus, 2017; Taylor *et al.,* 2017).

## Materials and methods

### Milkweed

This study was part of a larger project testing the effect of exposure to the neonicotinoid insecticide clothianidin on orientation of fall migratory monarch butterflies (Wilcox *et al.,* 2021). Swamp milkweed (*Asclepias incarnata*) was grown in commercial soil (LA4 Sunshine Loosefill, Sungro Horticulture, MA, USA) treated with either at 4, 8, 15 or 25 ng/g of clothianidin (neonicotinoid insecticide) or a control (i.e. distilled water) with 4 plants per 1.68 L (6 square inch) pot in environmental chambers at the University of Guelph Phytotron. However, we found no evidence of any measurable effect of neonicotinoid exposure on orientation for monarchs with early-life (caterpillar) exposure to clothianidin compared to controls, either when they were tested in a flight simulator or radio tracked in the wild (Wilcox *et al.,* 2021).

During plant growth, room temperature was set at 29°C during the day and 23°C at night, and they were exposed to a light intensity between 11914 and 16280 lx (18L:6D) based on Flockhart *et al.* (2012). Humidity was monitored hourly using a handheld thermohygrometer (Vaisala MI70 Measurement Indicator with HMP75 Humidity and Temperature Probe, Vaisala, Helsinki, Finland) with an average of 77% (SD ± 10%) RH. Plants were watered twice daily with reverse osmosis water until the soil was saturated and fertilized weekly with Plant-Prod Solutions fertilizer 17:5:17 NPK (Master Plant-Prod Inc., Brampton, Ontario, Canada). *Amblyseius swirskii* (Bioline AgroSciences Swirskiline Biocontrol Agent and Biobest Swirskii-Breeding-System) were introduced as a biocontrol measure to reduce the impact of thrips (Thysanoptera) (Flockhart **et al.*,* 2012).

### Capture and maintenance

We raised caterpillars from eggs laid by wild monarchs obtained from Gowanstown, Ontario (43.77°N, 80.91°W; male, *n* = 7; female, *n* = 13) on 14 August 2017 and the Guelph Lake Conservation Area (43.61°N, 80.26°W; male, *n* = 7; female, *n* = 11) from 2 to 6 August 2018. Wild monarch butterflies were held at ambient temperature in coin envelopes (6.35 $\times$ 10.8 cm) inside an animal carrier and humidity was maintained with a damp cloth at the bottom of the carrier to avoid the wings drying out during transport to the University of Guelph. Butterflies were weighed (Denver Instrument PI-602 scale, Denver Instrument, Bohemia, NY, USA) to the nearest 0.01 g. In order to maximize the number of eggs laid, wild monarchs were evenly distributed between large mesh enclosures (height $\times$ depth $\times$ width: 60 $\times$ 60 $\times$ 60 cm) for mating. The mesh enclosures, which contained only untreated milkweed at this stage of the experiment (grown in soil dosed with reverse osmosis water), were placed inside an incubator set at temperatures fluctuating between 29°C and 23°C with a light intensity between 11914 and 16290 lx (18L:6D) and an average of 77% (SD ± 10%) RH. An artificial nectar source (10% honey–water solution) was provided *ad libitum*.

In 2017 and 2018, we collected 192 eggs from the untreated milkweed plants per year by gently pressing a fine-tipped paintbrush along the edge of the egg and transferring to a milkweed leaf with latex holding the egg in place. Monarch caterpillars were reared in different types of enclosures in 2017 and 2018, but kept in the same environmental conditions until pupation. In 2017, monarch caterpillars were individually reared directly on the milkweed plants with pots enclosed with finely perforated mosquito netting (Bulk Mosquito Netting, CAT # 09A04.73, Lee Valley, Ottawa, Ontario, Canada). Light, temperature and humidity in a single chamber (area, 17.8 m^2^) in the University of Guelph Phytotron were maintained throughout monarch caterpillar development at ambient conditions during the early fall in Guelph, Ontario (43.5°N, 80.2°W; 13 hours light: 11 hours dark) at 21°C day:11°C night and an average of 87% (SD ± 6%) RH. Temperature did not decrease during development and these conditions are comparable to the settings used by Tenger-Trolander *et al.* (2019) to rear migratory monarchs (18°C with 14 hours light: 10 hours dark cycle). Caterpillars were fed milkweed *ad libitum* until pupation when chrysalids were then transferred to mesh enclosures (60 $\times$ 60 $\times$ 60 cm) separated by treatment after eclosion from 19 September to 3 October 2017. In 2018, leaves with a single egg were placed in separate large plastic containers and enclosed with finely perforated mosquito netting. At pupation, chrysalids were transferred to mesh enclosures (120 $\times$ 120 $\times$ 120 cm; Popadome Plant Dome, CAT # XC515, Lee Valley, Ottawa, Ontario, Canada) in the laboratory (ca. 19.5°C) where lighting cycle was variable and supplemented by negligible foyer lighting. Eclosion occurred from 14 to 19 September 2018. Adult monarchs were hand fed daily and provided dishes with a 10% honey–water solution within the enclosures (Flockhart *et al.,* 2012). We examined each individual for *Ophryocystis elektroscirrha* protozoan parasites by applying clear tape to the abdomen and analysing tape for spores under a microscope at 400× (Altizer and Oberhauser 1999) and, in contrast to Tenger-Trolander *et al.* (2019), infected butterflies were removed from the study to minimize impact of infection on flight. All procedures were conducted under the Ontario Ministry for Natural Resources Wildlife Scientific Collectors Permit (2017: #1086793; 2018: #1090000).

### Flight simulator testing

**Figure 1:**
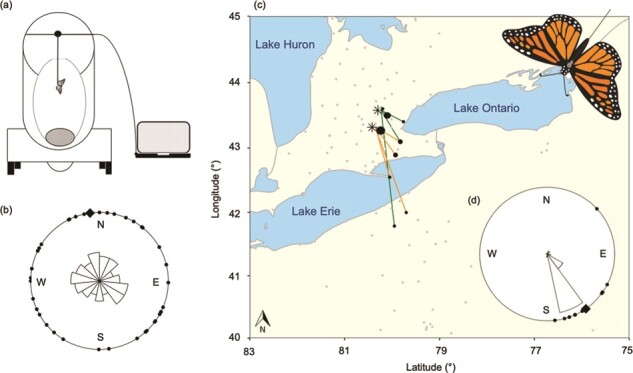
Orientation of captive-reared eastern North American migratory monarch butterflies (*D. plexippus*) (a) flown in a flight simulator that recorded (b) the direction of flight for individual monarchs (•) and group mean direction (◆) or (c) radio-tracked from Guelph (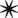, green lines) or Cambridge (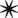, orange lines) to the first detection at a Motus tower (number of detections indicated by relative size of black dots and grey dots indicate active towers) with (d) the direction of flight displayed in a circular plot.

For a subset of monarch butterflies (control, *n* = 15; low dose, *n* = 16; high dose, *n* = 23; tested 2–5 days after eclosion), we assessed monarch orientation during the fall migratory period (17–23 September 2018) using flight simulators (Fig. 1a). Flight simulators were set up on the roof of the University of Guelph Phytotron, Guelph, Ontario and arranged so that no surrounding buildings could obstruct the view of individuals while in the flight cylinder (Mouritsen *et al.,* 2013). Tests occurred during daylight (09:30–16:00 EST) when the sun was fully visible from the simulator to ensure consistency of polarized light cues (Reppert *et al.,* 2004; Mouritsen *et al.,* 2013). Individual butterflies were tethered to an L-shaped rod (modified to ~2.5 cm; CAT # 718000, 0.05 cm $\times$ 15.2 cm Tungsten Rods, A-M Systems, WA, USA) inserted at the front of the dorsal thorax, avoiding flight muscle, and secured with super glue (All Purpose Krazy Glue No Run Gel, Elmer’s Products, High Point, NC, USA). Each tether was attached to a digital encoder that allowed 360° rotation and recorded orientation at 3° intervals and there was no detectable resistance in the tether that would bias the direction of flight. The encoder was adhered to a plexiglass rod supported within a large cylinder and attached to a nearby computer to record directional data (Mouritsen *et al.,* 2013). A fan at the base of the flight simulator provided airflow to encourage flight. Each monarch was flown once for 12 minutes (5 direction recordings/sec), with the initial 2 minutes for acclimation and to minimize the impacts of stress-induced unidirectional flight response (Perez *et al.,* 1999). Monarchs were removed from the study (control, *n* = 3; low dose, *n* = 7; high dose, *n* = 5) if they did not show a characteristic pattern of flight (i.e. strong flapping with intermittent gliding).

### Radio-telemetry tracking

From a subset of monarchs raised in captivity, we tracked individuals using radio transmitters during early migration. Monarchs were outfitted with 200 mg NanoTags (Lotek Wireless Fish & Wildlife Monitoring, Newmarket, Ontario, Canada), each programmed to emit unique 166.380 MHz pulses every 4.7 seconds to maximize the probability of detection and allow individual identification (i.e. higher rate of pulses gives a greater temporal resolution, but shorter tag lifespan; Taylor *et al.,* 2017). Large individuals (>0.3 g) were selected to minimize weight restrictions imposed by the tags and maximize the capacity for long-distance flight. On 5 October 2017, 41 monarch butterflies (control, *n* = 12; low dose, *n* = 10; high dose, *n* = 19) were released in an open field in Guelph, Ontario (43.57°N, 80.23°W), centered between adjacent Motus towers. On 27 September 2018, 43 monarchs (control, *n* = 14; low dose, *n* = 14; high dose, *n* = 15) were released on a hill, above the tree line, at the base of the RARE Charitable Research Reserve (https://raresites.org/) Motus tower (43.38°N, 80.35°W) in Cambridge, Ontario. Radio telemetry requires that the signal emitted by the tag is received by a radio tower to identify the location of the monarchs during migration (Taylor *et al.,* 2017). When the radio transmitter is close to the ground (as opposed to at a high altitude), radio towers can receive signals from 500 m up to 3 km depending on the type, height, orientation of the antennae, topography, habitat structure and nearby human-made structures (Taylor *et al.,* 2017). The limited detection range of the towers contributed to only a fraction of the released monarchs being detected by towers other than the tower located at the release site (see below). Moreover, in the wild, radio transmitters had a limited battery lifespan (1–16 days, mean = 4 days), meaning that some monarchs may have not been detected at towers because tag transmission had ceased. At the time of release, the Motus telemetry array consisted of more than 100 independent very high frequency telemetry towers across southern Ontario and the northern United States, with towers in all directions around the sites of release (Motus, 2017; Taylor *et al.,* 2017). False detections were removed from analysis following the procedures outlined by Crewe *et al.* (2019). More specifically, we ran preliminary filters to remove detections with run lengths (i.e. number of detections) of <2 and false detections as a result of noise (e.g. detections prior to release or beyond the species range, towers recording spurious detections). We also examined ambiguous detections manually, using contextual information to identify true detections (Crewe *et al.,* 2019); for instance, removing detections that bounced between multiple towers located several hundred kms or more apart. We also removed detections recorded on the day of release at adjacent towers with overlapping detection ranges with the release site to avoid inaccurately assigning a direction of flight when the monarchs had not left the area. This removal and data filtering outlined above resulted in true detections for 9 monarch butterflies in 2017 (22% of total released) and 20 monarchs in 2018 (47% of total released; see Supplementary Material Table S2 for more information).

### Statistical analysis

North American migratory monarch butterflies are expected to orient in a southward direction when flown in a flight simulator (Froy *et al.,* 2003; Guerra and Reppert, 2013). We calculated the mean direction (0° to 359°) and vector strength (r: 0–1) for each monarch butterfly flight using the program Oriana version 4.02 (Mouritsen and Frost, 2012). Vector strength is a measure of concentration for circular data with high values indicating a tighter grouping around the mean direction (Mouritsen *et al.,* 2013; Kovach Computing Services, 2020).

We then calculated the mean group direction and vector strength separately for monarchs flown in the flight simulator and radio-tracked using the Motus telemetry using Oriana. We also ran a Rayleigh test to assess the significance of the vector strength, allowing us to determine if monarchs showed directional unimodal flight and calculated the percentage of individual monarch butterflies that flew in the expected southward direction.

We calculated Spearman’s Rank correlation coefficients with the *stats* package (v3.6.2) in R version 3.4.1 (R Core Team, 2015) to assess the relationship between distance travelled and time (i.e. greater distance travelled with a longer duration of time since release). To understand the effect of wind on migratory direction and the year of testing on flight direction, we ran two models using the *circular* package (v0.4-93, Lund *et al.,* 2017). Maximum wind speed (km h^−1^) was included as the predictor with direction of flight (degrees) as a response variable in a circular-linear model. Data on wind speed were obtained from Environment and Climate Change Canada (2019) for weather stations closest to the Motus towers (1.7–15.9 km) from where monarchs were first detected, with the exception of 8 stations where data were unavailable or missing. Monarchs released in 2017 were radio tracked later in the fall compared to 2018, so to determine whether the year of testing influenced migratory flight, we ran a circular ANOVA with year as the predictor variable and direction of flight (degrees) as a response variable.

## Results

Although the mean direction for monarchs flown in the flight simulator was σ = 352° (N), individuals showed strong orientation in a variety of directions, resulting in the sample only being weakly concentrated around the mean (*n* = 39, *r* = 0.07, Rayleigh test, *z* = 0.2, *P* = 0.82; Fig. 1b). Only 26% of monarchs tested in the flight simulator oriented in a southeast to southwest direction (10/39; Supplementary Material Table S1). In contrast, 97% of radio-tracked monarchs (28/29) flew south to southeast (σ = 147°; Fig. 1c and d; Supplementary Material Table S2). In contrast to monarchs tested in the flight simulator, the direction of flight for radio-tracked monarchs was strongly concentrated around the mean (*n* = 29, *r* = 0.93, Rayleigh test, *z* = 24.93, *P* < 0.001; Fig. 1d). Monarchs were first detected 1–16 days after release (Supplementary Material Table S2) at towers ranging from 12 km (52%, 15/29) up to ~200 km (3%, 1/29) from the release site. The number of days to first detection was correlated with distance from the release site (Spearman’s rank correlation, *n* = 29, *r*_s_ = 0.70, *P* < 0.001). There was no evidence for an effect of wind speed on flight direction (Table 1), but there was evidence that year of testing influenced flight direction (Table 1), with monarchs released in 2017 tending to orient south-southeast and monarchs released in 2018 tending to orient southeast.

**Table 1:** Eastern North American migratory monarch butterflies (*D. plexippus*) were reared in environmental chambers simulating autumn conditions until pupation, and then radio tracked during fall migration

Wind speed						
Est.	Std. Error	t	p	logLik	μ	κ
−0.0005	0.0006	0.70	0.24	37.04	289.7	15.25
Year of testing						
	DF	SS	MS	F	p	
	1	0.75	0.75	15.18	0.0006	

In a circular-linear model, maximum wind speed (km h^−1^) influenced direction of flight (degrees; *P* < 0.05). In a separate circular-ANOVA model, there was evidence that year also influenced the direction of flight (degrees; *P* < 0.05).

## Discussion

Our results provide evidence that monarch butterflies raised in captivity in a controlled laboratory environment, but later exposed to natural conditions (including sunlight and photoperiod), can calibrate the mechanism governing directional flight, allowing them to properly orient southward towards Mexico after they are released into the wild. Monarch butterflies tested in the flight simulator flew in all directions and only 10 of 39 (26%) individuals flew in the southeast to southwest direction. However, when released into natural conditions, 97% of free-flying monarchs that could be reliably detected by automated towers flew in the south to southeast direction. We also have no *a priori* reason to expect differences in flight direction between reliably detected monarchs and those which could not be detected reliably (e.g. Motus tower coverage was relatively even in all directions around the release site). Thus, while our study confirms the results from Tenger-Trolander *et al.* (2019) that most captive-reared monarchs tested in a flight simulator do not show proper orientation towards their Mexican wintering grounds, we also demonstrate that monarchs released in the wild are capable of calibrating their orientation mechanisms responsible for directional flight. Therefore, our results provide support that migratory flight is not impacted when monarchs are reared under conditions approximating those encountered in the wild. Though the environmental conditions in this experiment may differ for monarchs reared by hobbyists, our results suggest that under certain conditions (e.g. releasing first-generation offspring from wild-caught parents), captive rearing could help supplement monarch populations and remains a valuable educational tool for highlighting the natural history and biology of butterflies.

The results of our experiment suggest that outdoor environmental conditions are required for proper directional flight during migration. The sun’s position in the sky may act as a cue for the direction of migratory flight (Taylor *et al.*, 2019). Sunlight cues are perceived through the eyes and monarchs also have a light-sensitive molecular clock in their antennae (Reppert and Weaver, 2002; Reppert, 2006; Merlin *et al.,* 2009; Guerra *et al.,* 2012), with information from these two systems likely integrated in the midbrain (Reppert *et al.,* 2010). Disruption of this molecular mechanism by restricting natural light results in disoriented flight (Merlin *et al.,* 2009; Guerra *et al.,* 2012), providing evidence that sunlight is required for monarchs to calibrate flight orientation. A similar calibration with environmental cues was found in *Catharus* thrushes (Cochran *et al.,* 2004). After exposure to experimental magnetic fields, grey-cheeked thrushes (*Catharus minimus*) and Swainson’s thrush (*Catharus ustulatus*) were released and their flight patterns tracked using radio transmitters (Cochran *et al.,* 2004). On the first night, birds flew westward, but corrected their orientation by the second night after they were exposed to ‘normal’ twilight cues and flew in the proper northward direction (Cochran *et al.,* 2004). Though mechanisms underpinning flight orientation differ between birds and insects (Cochran *et al.,* 2004; Mouritsen, 2018), it is possible that monarchs can also calibrate the direction of flight using information obtained via skylight and other natural cues. In particular, ultraviolet (UV) light, notably UVA, is needed for proper monarch orientation (Froy *et al.,* 2003; Guerra *et al.,* 2014). Tenger-Trolander and Kronfrost (2020) reared monarchs indoors with exposure to sunlight, but light was filtered through glass windows. Glass can reduce UVA and UVB transmission (Tuchinda *et al.,* 2006), though the degree of photoprotection depends on glass type, colour and coating (Duarte *et al.,* 2009). So, it is unsurprising that monarchs raised indoors with exposure to sunlight through glass did not consistently orient south in subsequent flight simulation tests as observed in this study (see also Tenger-Trolander and Kronfrost, 2020). Alternatively, differences in the photoperiod between our study and the study by Tenger-Trolander *et al.* (2019) could underlie the observed differences in orientation. We used a slightly shorter photoperiod to approximate conditions experienced by monarchs in fall. Photoperiod is a critical cue for animal migration and, in some species, has been implicated in triggering the change in migratory direction (Dingle, 2014). Therefore, it is also possible that this variation in photoperiod is required for monarch butterflies to orient in a southward direction during fall migration.

Captive rearing of monarch butterflies for wildlife education, breeding programs or by hobbyists can enhance conservation efforts if precautions are taken to rear monarchs in conditions that allow exposure to natural environmental conditions. Though commercially reared monarchs tested by Tenger-Trolander *et al.* (2019) showed a random orientation, the authors contrast their findings with a successful tag and release by Maeckle (2018) where released monarchs were re-sighted in Mexico and our results clearly demonstrate that upon release monarchs regain proper orientation. Moreover, a recent study showed that indoor-reared monarchs can successfully reach their overwintering sites when exposed to controlled temperatures and natural sunlight (James and Kappan, 2021). Therefore, we suggest that under controlled rearing conditions, with exposure to natural sunlight, loss of orientation capacity may be negligible and future studies should determine the minimum duration of sunlight required to establish southward directional flight. Though the practice of captive rearing is contentious due to the potential for disease transmission (Journey North, 2015; Monarch Joint Venture, 2018), morphological and physiological changes (Tenger-Trolander *et al.,* 2019; Davis *et al.,* 2020) and concerns around genetic viability (Journey North, 2015; Willoughby *et al.,* 2017; Monarch Joint Venture, 2018) when these risks are minimized, reintroduction of monarch butterflies to the wild could contribute towards reversing the declines of migratory populations. More work would be needed, however, to determine the optimal rearing and environmental conditions for captive rearing and release. Captive rearing of monarchs is not only a tool for conservation, but is also an extraordinary educational opportunity for the public to interact with nature and engage in conservation. The incredible social appeal of monarch butterflies and captive rearing for educational purposes encourages interactions between the public, educators and scientists (Gustafsson *et al.,* 2015). Thus, under proper conditions, captive rearing offers an opportunity for the public to engage in the conservation of this beloved and iconic species.

We acknowledge that captive-reared monarchs tested for flight direction in either the flight simulator or via radio-tracking lacked a proper comparison with wild-caught ‘controls’. However, expected southward flight directions of monarchs tested in both the flight simulator and via radio tracking were based on previously published studies. From September to October, Mouritsen *et al*. (2013) found that wild caught monarchs tested in a flight simulator in Guelph, Ontario (the same location where our study was conducted) oriented southeast to southwest. More recently, Knight *et al*. (2019) provided evidence that wild-caught monarchs (*n* = 43) captured on the Bruce Peninsula (~250 km north of Guelph) radio tracked in September oriented southeast prior to crossing Lake Erie. Thus, while our study lacks comparisons to wild-caught monarchs, we are confident that expected (natural) flight direction from these two methods remains valid.

A number of other caveats to our study design and conclusions should be noted. First, we suspect that the small difference found in flight direction between years was likely due to the date of eclosion of the tested butterflies, with monarchs in 2017 having eclosed later in fall (23 September–1 October 2017) than monarchs from 2018 (14–19 September 2018). It is also possible that environmental cues known to trigger a migratory generation, such as decreasing day length and temperature (Goehring and Oberhauser, 2002), could also influence flight orientation. However, our experimental design did not allow us to investigate the potential effect of changing environmental conditions on flight direction. We were also unable to determine the duration of exposure to solar cues required for calibration of the molecular clock mechanism. Nor were we able to test individual monarch butterflies in the flight simulator and then release the same individuals in the wild. Monarchs tested in the flight simulator were temporarily compromised due to the insertion of a rod into the front of the dorsal thorax and showed visible signs of exhaustion (e.g. lethargy) after testing. With the continued development of tracking technology, it is likely that we will soon have the ability to track monarchs and other insects at finer spatial resolutions and over multiple days during their migratory journey. When that occurs, our understanding of the proximate mechanisms that govern orientation and effects of captive rearing will likely improve.

The declines of Eastern North American monarch butterflies over the past two decades (Thogmartin *et al.,* 2017) serve as a reminder of the challenges faced in conserving biodiversity, particularly of insects, and in the conservation of this species-at-risk. Moreover, with increasing awareness of numerous threats to monarch butterfly populations (Flockhart *et al*., 2015; Thogmartin *et al.,* 2017), extensive support has been garnered across Canada, the USA and Mexico for monarch conservation. Our results confirm studies on the impact of captive rearing on monarch butterflies, but only when monarchs are tested in a confined flight simulator (Tenger-Trolander *et al.,* 2019). Captive-reared monarchs gain proper flight orientation when released into the wild, demonstrating that the popular activity of rearing monarch butterflies from caterpillars in captivity could still be a viable conservation tool and important education element to conserve both monarchs and other butterfly species, at least under the context of certain rearing conditions that were followed in this study. Whether monarchs reared under different conditions from this study show the same flight orientation remains to be tested.

## Supplementary Material

Data supporting the analysis is included as part of the Supplementary Material. Motus data is available at https://motus.org/data/downloads (Project ID # 209).

## Acknowledgements

We thank Taylor Van Belleghem, Angela Demarse and Samantha Knight, as well as our team of volunteers, for assistance with data collection. Thank you to Mike Mucci and Tannis Slimmon for technical support and coordinating use of the University of Guelph Phytotron, as well as to Stuart MacKenzie for technical support during use of the Motus telemetry system. Jenna Quinn (rare Charitable Research Reserve), Mike vandenTillaart (Lotek Wireless Inc.) and Dave Gambin assisted with the monarch release in Cambridge, Ontario.

## Funding

This work was supported by a Natural Sciences and Engineering Research Council (NSERC) Discovery Grant to D.R.N. and N.E.R. (2015-06783), the Food from Thought: Agricultural Systems for a Healthy Planet Initiative, by the Canada First Research Excellence Fund (grant 000054) and a grant from the Ontario Ministry of Agriculture, Food and Rural Affairs to A.E.M.N and D.R.N (030267). Funding was also provided by Environment and Climate Change Canada to G.W.M. An NSERC Alexander Graham Bell Canada Graduate Scholarship (CGS D) and Ontario Graduate Scholarship provided support for A.A.E.W. N.E.R. is supported as the Rebanks Family Chair in Pollinator Conservation by the Weston Family Foundation.

## Author contributions

A.A.E.W., A.E.M.N., N.E.R. G.W.M. and D.R.N. conceived and designed the project. A.A.E.W. conducted the experimental work, analysed the data and drafted the original manuscript. All authors contributed to writing and revising the manuscript.

## Supplementary Material

Wilcox_et_al_CONPHYS-2020-159_Suppl_(08_Apr_21)_coab032

## References

[ref1] Altizer SM, Oberhauser KS (1999) Effects of the protozoan parasite *Ophryocystis elektroscirrha* on the fitness of monarch butterflies (*Danaus plexippus)*. *J Invert Pathol* 74: 76–88. doi: 10.1006/jipa.1999.4853. 10388550

[ref2] Bauman K, Blumer E, Crosier A, Fallon J, Geise G, Grisham J, Ivy J, Long S, Rogers A, Schwartz K, et al. (2010) Global cheetah *ex situ* planning: linking managed populations working group. CBSG News 21:1–4.

[ref3] Birkett LP, Newton-Fisher NE (2011) How abnormal is the behaviour of captive, zoo-living chimpanzees? *PLoS One* 6: e20101. doi: 10.1371/journal.pone.0020101. 21698219 PMC3116814

[ref4] Blanchard RJ, Flannelly KJ, Blanchard DC (1986) Defensive behaviors of laboratory and wild *Rattus norvegicus*. *J Comp Psychol* 100: 101–107. doi: 10.1037/0735-7036.100.2.101. 3720282

[ref5] Brower LP (1995) Understanding and misunderstanding the migration of the monarch butterfly (Nymphalidae) in North America: 1857-1995. *J Lepid Soc* 49: 304–385.

[ref6] Brower LP, Taylor OR, Williams EH, Slayback DA, Zubieta RR, Ramírez MI (2012) Decline of monarch butterflies overwintering in Mexico: is the migratory phenomenon at risk? Insect Conserv Divers 5: 95–100. doi: 10.1111/j.1752-4598.2011.00142.x.

[ref7] Chamove AS, Hosey GS, Schaetzel P (1988) Visitors excite primates in zoos. *Zoo Biol* 7: 359–369. doi: 10.1002/zoo.1430070407.

[ref8] Cochran WW, Mouritsen H, Wikelski M (2004) Migrating songbirds recalibrate their magnetic compass daily from twilight cues. *Science* 304: 405–408. doi: 10.1126/science.1095844. 15087541

[ref9] Crewe TL, Crysler Z, Taylor P (2019) Data cleaning. Motus R Book: A Walk Through the Use of R for Motus Automated Radio-Telemetry Data, https://motus.org/MotusRBook/(last accessed 30 September 2020).

[ref10] Davis AK, Smith FM, Ballew AM (2020) A poor substitute for the real thing: captive-reared monarch butterflies are weaker, paler and have less elongated wings than wild migrants. *Biol Lett* 16: 20190922. doi: 10.1098/rsbl.2019.0922. 32264783 PMC7211457

[ref11] Dingle H (2014) Orientation and navigation. In Migration: The Biology of Life on the Move. Oxford University Press, Oxford, England, pp. 135–159.

[ref12] Duarte I, Rotter A, Malvestiti A, Silva M (2009) The role of glass as a barrier against the transmission of ultraviolet radiation: an experimental study. Photodermatol Photoimmunol Photomed 25: 181–184. doi: 10.1111/j.1600-0781.2009.00434.x. 19614895

[ref13] Environment and Climate Change Canada (2019) Historical data. https://climate.weather.gc.ca/historical_data/search_historic_data_e.html(last accessed 30 September 2020).

[ref14] Fisher DN, James A, Rodríguez-Muñoz R, Tregenza T (2015) Behaviour in captivity predicts some aspects of natural behaviour, but not others, in a wild cricket population. *Proc R Soc B* 282: 20150708. doi: 10.1098/rspb.2015.0708. PMC4590455 26019161

[ref15] Flockhart DTT, Martin TG, Norris DR (2012) Experimental examination of intraspecific density-dependent competition during the breeding period in monarch butterflies (*Danaus plexippus*). *PLoS One* 7: e45080. doi: 10.1371/journal.pone.0045080. 22984614 PMC3440312

[ref62] Flockhart DTT, Pichancourt J-B, Norris DR, Martin TG (2015) Unravelling the annual cycle in a migratory animal: breeding?season habitat loss drives population declines of monarch butterflies. J Anim Ecol 84: 155–165. doi: 10.1111/1365-2656.12253. 24903085

[ref16] Froy O, Gotter AL, Casselman AL, Reppert SM (2003) Illuminating the circadian clock in monarch butterfly migration. *Science* 300: 1303–1305. doi: 10.1126/science.1084874. 12764200

[ref17] Goehring L, Oberhauser KS (2002) Effects of photoperiod, temperature, and host plant age on induction of reproductive diapause and development time in *Danaus plexippus*. *Ecol Entomol* 27: 674–685. doi: 10.1046/j.1365-2311.2002.00454.x.

[ref18] Gro Vea Salvanes A, Braithwaite V (2006) The need to understand the behaviour of fish reared for mariculture or restocking. *ICES J Mar Sci* 63: 345–354. doi: 10.1016/j.icesjms.2005.11.010.

[ref19] Guerra PA, Merlin C, Gegear RJ, Reppert SM (2012) Discordant timing between antennae disrupts sun compass orientation in migratory monarch butterflies. *Nat Commun* 3: 958. doi: 10.1038/ncomms1965. 22805565 PMC3962218

[ref20] Guerra PA, Reppert SM (2013) Coldness triggers northward flight in remigrant monarch butterflies. *Curr Biol* 23: 419–423. doi: 10.1016j.cub.2013.01.052. 23434279 10.1016/j.cub.2013.01.052

[ref21] Guerra PA, Gegear RJ, Reppert SM (2014) A magnetic compass aids monarch butterfly migration. *Nat Commun* 5: 4164. doi: 10.1038/ncomms5164. 24960099 PMC4090716

[ref22] Gustafsson KM, Agrawal AA, Lewenstein BV, Wolf SA (2015) The monarch butterfly through time and space: the social construction of an icon. *Bioscience* 65: 612–622. doi: 10.1093/biosci/biv045.

[ref23] Howard E, Davis AK (2004) Documenting the spring movements of monarch butterflies with Journey North, a citizen science program. In MS Oberhauser, MJ Solensky, eds, The Monarch Butterfly: Biology and Conservation. Cornell University Press, United States of America, pp 105–114.

[ref24] Hughes DG, Bennett PM (1991) Captive breeding and the conservation of invertebrates. *Int Zoo Yearb* 30: 45–51. doi: 10.1111/j.1748-1090.1991.tb03464.x.

[ref25] Ings TC, Raine NE, Chittka L (2009) A population comparison of the strength and persistence of innate colour preference and learning speed in the bumblebee *Bombus terrestris*. *Behav Ecol Sociobiol* 63:1207–1218. doi: 10.1007/s00265-009-0731-8.

[ref26] James DG, Kappan L (2021) Further insights on the migration biology of monarch butterflies, *Danaus plexippus* (Lepidoptera: Nymphalidae) from the Pacific Northwest. *Insects* 12: 161. doi: 10.3390/insects12020161. 33672834 PMC7917764

[ref27] Journey North (2015) Captive breeding and releasing monarchs. https://journeynorth.org/tm/monarch/conservation_action_release.pdf (last accessed 30 September 2020).

[ref28] Knight SM, Pitman GM, Flockhart DTT, Norris DR (2019) Radio-tracking reveals how wind and temperature influence the pace of daytime insect migration. Biol Lett 15: 20190327. doi: 10.1098/rsbl.2019.0327. 31266418 PMC6684972

[ref29] Kountoupes DL, Oberhauser KS (2008) Citizen science and youth audiences: educational outcomes of the Monarch Larva Monitoring Project. *J Commun Engage Scholar* 1: 5 https://digitalcommons.northgeorgia.edu/jces/vol1/iss1/5 (last accessed 07 October 2020).

[ref30] Kovach Computing Services (2020) Oriana. Version 4.02. http://kovcomp.co.uk (last accessed 30 September 2020).

[ref31] Lund U, Agostinelli C, Arai H, Gagliardi A, Portugues EG, Giunchi D, Irisson J-O, Pocernich M, Rotolo F (2017) circular: Circular statistics. Version 0.4-93. http://cran.r-project.org/web/packages/circular/ (last accessed 30 September 2020).

[ref32] Maeckle M (2018) Five monarch butterflies tagged and released at San Antonio Festival made it to Mexico. https://texasbutterflyranch.com/2018/04/25/five-monarch-butterflies-tagged-and-released-at-san-antonio-festival-made-it-to-mexico/ (last accessed 30 September 2020).

[ref33] Mason GJ (2010) Species differences in responses to captivity: stress, welfare and the comparative method. *Trends Ecol Evol* 25: 713–721. doi: 10.1016/j.tree.2010.08.011. 20952089

[ref34] McPhee ME (2004) Generations in captivity increases behavioral variance: considerations for captive breeding and reintroduction programs. *Biol Conserv* 115: 71–77. doi: 10.1016/S0006-3207(03)00095-8.

[ref35] Merlin C, Gegear RJ, Reppert SM (2009) Antennal circadian clocks coordinate sun compass orientation in migratory monarch butterflies. *Science* 325:1700–1704. doi: 10.1126/science.1176221. PMC2754321 19779201

[ref36] Mettke C (1995) Ecology and environmental enrichment – the example of parrots. In: U Gansloßer, JK Hodges, W Kaumanns, eds, Research and Captive Propagation. Filander, Fürth Germany, pp 257–262.

[ref37] Monarch Joint Venture. 2018. Raising monarchs: why or why not? (Revised handout). https://monarchjointventure.org/blog/revised-handout-raising-monarchs-why-or-why-not (last accessed 30 September 2020).

[ref38] Motus (Motus Wildlife Tracking System) (2017) About Motus. http://motus.org/about (last accessed 30 September 2020).

[ref39] Mouritsen H (2018) Long-distance navigation and magnetoreception in migratory animals. *Nature* 558: 50–59. doi: 10.1038/s41586-018-0176-1. 29875486

[ref40] Mouritsen H, Derbyshire R, Stalleicken J, Mouritsen OØ, Frost BJ, Norris DR (2013) An experimental displacement and over 50 years of tag-recoveries show that monarch butterflies are not true navigators. *Proc Natl Acad Sci U S A* 110:7348–7353. doi: 10.1073/pnas.1221701110. PMC3645515 23569228

[ref41] Mouritsen H, Frost BJ (2012) Virtual migration in tethered flying monarch butterflies reveals their orientation mechanisms. *Proc Natl Acad Sci U S A* 99: 10162–10166. doi: 10.1073/pnas.152137299. PMC126641 12107283

[ref42] Oberhauser KS, Prysby MD (2008) Citizen science: creating a research army for conservation. *Am Entomol* 54: 103–105. doi: 10.1093/ae/54.2.103.

[ref43] Perez SM, Taylor OR, Jander R (1999) The effect of a strong magnetic field on monarch butterfly (*Danaus plexippus*) migratory behaviour. *Naturwiss* 86: 140–143. doi: 10.1007/s001140050587.

[ref44] Pleasants J, Oberhauser KS (2012) Milkweed loss in agricultural fields because of herbicide use: effect on the monarch butterfly population. Insect Conserv Divers 6: 135–144. doi: 10.1111/j.1752-4598.2012.00196.x.

[ref45] Pleasants JM (2016) Milkweed restoration in the Midwest for monarch butterfly recovery: estimates of milkweeds lost, milkweeds remaining and milkweeds that must be added to increase the monarch population. Insect Conserv Divers 10: 42–53. doi: 10.1111/icad.12198.

[ref46] R Core Team (2015) R: a language and environment for statistical computing. https://www.r-project.org/ (last accessed 30 September 2020).

[ref47] Reppert SM (2006) A colorful model of the circadian clock. *Cell* 124: 233–236. doi: 10.1016/j.cell.2006.01.009. 16439193

[ref48] Reppert SM, Gegear RJ, Merlin C (2010) Navigational mechanisms of migrating monarch butterflies. *Trends Neurosci* 33: 399–406. doi: 10.1016/j.tins.2010.04.004. 20627420 PMC2929297

[ref49] Reppert SM, Weaver DR (2002) Coordination of circadian timing in mammals. *Nature* 418: 935–941. doi: 10.1038/nature00965. 12198538

[ref50] Reppert SM, Zhu H, White RH (2004) Polarized light helps monarch butterflies navigate. *Curr Biol* 14: 155–158. doi: 10.1016/j.cub.2003.12.034. 14738739

[ref51] Ries L, Oberhauser K (2015) A citizen army for science: quantifying the contributions of citizen scientists to our understanding of monarch butterfly biology. *Bioscience* 65: 419–430. doi: 10.1093/biosci/biv011.

[ref52] Taylor OR Jr, Lovett JP, Gibo DL, Weiser EL, Thogmartin WE, Semmens DJ, Diffendorfer JE, Pleasants JM, Pecoraro SD, Grundel R (2019) Is the timing, pace, and success of the monarch migration associated with sun angle? *Front Ecol Evol* 7: 442. doi: 10.3389/fevo.2019.00442.

[ref53] Taylor PD, Crewe TL, Mackenzie SA, Lepage D, Aubry Y, Crysler Z, Finney G, Francis CM, Guglielmo CG, Hamilton DJ et al. (2017) The Motus Wildlife Tracking System: a collaborative research network to enhance the understanding of wildlife movement. *Avian Conserv Ecol* 18: 8. doi: 10.5751/ACE-00953-120108.

[ref54] Tenger-Trolander A, Lu W, Noyes M, Kronfrost MR (2019) Contemporary loss of migration in monarch butterflies. *Proc Natl Acad Sci U S A* 116: 14671–14676. doi: 10.1073/pnas.1904690116. 31235586 PMC6642386

[ref55] Tenger-Trolander A, Kronfrost MR (2020) Migration behaviour of commercial monarchs reared outdoors and wild-derived monarchs reared indoors. *Proc R Soc B* 287: 20201326. doi: 10.1098/rspb.2020.1326. PMC7575518 32752991

[ref56] Thogmartin WE, Wiederholt R, Oberhauser K, Drum RG, Diffendorfer JE, Altizer S, Taylor OR, Pleasants J, Semmens D, Semmens B et al. (2017) Monarch butterfly population decline in North America: identifying the threatening processes. *R Soc Open Sci* 4: 170760. doi: 10.1098/rsos.170760. 28989778 PMC5627118

[ref57] Tuchinda C, Srivannaboon S, Lim HW (2006) Photoprotection by window glass, automobile glass, and sunglasses. *J Am Acad Dermatol* 54: 845–854. doi: 10.1016/j.jaad.2005.11.1082. 16635665

[ref58] Urquhart FA (1960) Migration. In The Monarch Butterfly. University of Toronto Press, Toronto, Canada, pp. 77–94

[ref59] Urquhart FA, Urquhart NR (1978) Autumnal migration routes of the eastern population of the monarch butterfly (*Danaus p. plexippus* L.; Danaidea; Lepidoptera) in North America to the overwintering site in the Neovolcanic Plateau of Mexico. *Can J Zool* 56:1759–1764. doi: 10.1139/z78-240.

[ref60] Wilcox AAE, Newman AEM, Raine NE, Mitchell GW, Norris DR (2021) Effects of early-life exposure to sublethal levels of a common neonicotinoid insecticide on the orientation and migration of monarch butterflies (*Danaus plexippus*). J Exp Biol 224: jeb230870 doi:10.1242/jeb.230870. 33334898

[ref61] Willoughby JR, Ivy JA, Lacy RC, Doyle JM, DeWoody JA (2017) Inbreeding and selection shape genomic diversity in captive populations: implications for the conservation of endangered species. *PLoS One* 12: e0175996. doi: 10.1371/journal.pone.0175996. 28423000 PMC5396937

