# Supplementary material for: Captive-reared migratory monarch butterflies show natural orientation when released in the wild
Source: Conserv Physiol. 2021 May 11;9(1):coab032. doi: 10.1093/conphys/coab032 (PMC8355447; doi:10.1093/conphys/coab032)
Supplement: Wilcox_et_al_CONPHYS-2020-159_Suppl_(08_Apr_21)_coab032 [file wilcox_et_al_conphys-2020-159_suppl_(08_apr_21)_coab032.docx]

# Supplementary material

**Captive-reared migratory monarch butterflies show natural orientation when released in the wild**

**Alana A. E. Wilcox**^1*^**, Amy E. M. Newman**^1^**, Nigel, E. Raine**^2^**, Greg W. Mitchell^3,4^, and D. Ryan Norris**^1,5^

*^1^ Department of Integrative Biology, University of Guelph, Guelph, ON, N1G 2W1, Canada*

*^2^ School of Environmental Sciences, University of Guelph, Guelph, ON, N1G 2W1, Canada*

*^3^ Wildlife Research Division, Environment and Climate Change Canada, National Wildlife Research Centre, 1125 Colonel By Drive, Ottawa, ON, K1S 5B6, Canada*

*^4^ Department of Biology, Carleton University, 1125 Colonel By Drive, Ottawa, ON, K1S 5B6, Canada*

^5^ *Nature Conservancy of Canada, 245 Eglington Avenue East, Toronto, ON, M4P 3J1, Canada*

* To whom correspondence should be addressed: [alanaaewilcox@gmail.com](mailto:alanaaewilcox@gmail.com)

## Supplementary Tables

**Table S1.** Eastern North American migratory monarch butterflies (*Danaus plexippus*) reared in environmental chambers simulating autumn conditions (day: 29°C, night 23°C and 77% RH) until pupation. Monarchs were reared on swamp milkweed (*Asclepias incarnata*) grown in commercial soil treated with either at 15 or 25 ng/g of clothianidin (neonicotinoid: CLO) insecticide or a control (0 ng/g of clothianidin) before being tested in an outdoor flight simulator that recorded orientation between 0° to 359° (n = 39). The mean direction (° ± circular SD) and vector strength (r), representing the concentration of the data between 0 (data are evenly spread) to 1 (data are concentrated around the mean), was calculated for each individual. Rayleigh tests were used to determine whether monarchs showed directional flight (p < 0.05); however, the large sample size (n = 3000) from the flight simulator tests results in low p values without practical significance. Each row represents an individual monarch butterfly.

## CLO

**Treatment (ng/g)**

**Cardinal direction**

**Mean direction**

**Flight simulator**

**Circular SD (°)**

|  | **(°)** |  | | |
| --- | --- | --- | --- | --- |
| 15 E | 91 | 36 | 0.82 | 2030 < 0.001 |
| 0 ENE | 76 | 51 | 0.68 | 1367 < 0.001 |
| 25 ENE | 60 | 57 | 0.61 | 1104 < 0.001 |
| 25 ESE | 119 | 74 | 0.43 | 561 < 0.001 |
| 25 ESE | 117 | 56 | 0.62 | 1168 < 0.001 |
| 25 ESE | 117 | 75 | 0.42 | 537 < 0.001 |
| 0 ESE | 112 | 21 | 0.93 | 2613 < 0.001 |
| 25 N | 352 | 42 | 0.76 | 1735 < 0.001 |
| 0 N | 12 | 90 | 0.29 | 250 < 0.001 |
| 25 N | 6 | 41 | 0.78 | 1820 < 0.001 |
| 0 N | 0 | 0 | 1.00 | 3000 < 0.001 |
| 25 NE | 39 | 79 | 0.38 | 441 < 0.001 |
| 25 NNE | 38 | 93 | 0.27 | 211 < 0.001 |
| 0 NNE | 30 | 124 | 0.10 | 28 < 0.001 |
| 15 NNE | 23 | 144 | 0.04 | 6 0.004 |
| 15 NNW | 347 | 13 | 0.98 | 2857 < 0.001 |
| 0 NNW | 340 | 13 | 0.97 | 2841 < 0.001 |

**Rayleigh test**

**r z p**

| 25 NNW | 334 | 95 | 0.25 | 193 < 0.001 |
| --- | --- | --- | --- | --- |
| 25 NNW | 333 | 91 | 0.29 | 245 < 0.001 |
| 15 S | 181 | 142 | 0.05 | 7 0.001 |
| 25 S | 172 | 41 | 0.78 | 1805 < 0.001 |
| 25 SE | 138 | 76 | 0.42 | 522 < 0.001 |
| 15 SE | 137 | 57 | 0.61 | 1128 < 0.001 |
| 15 SE | 127 | 43 | 0.75 | 1706 < 0.001 |
| 0 SE | 126 | 105 | 0.19 | 105 < 0.001 |
| 0 SSE | 147 | 77 | 0.41 | 498 < 0.001 |
| 25 SSW | 212 | 153 | 0.03 | 2 0.08 |
| 25 SW | 230 | 1 | 1.00 | 2998 < 0.001 |
| 0 SW | 221 | 42 | 0.76 | 1734 < 0.001 |
| 25 W | 280 | 68 | 0.50 | 740 < 0.001 |
| 0 W | 273 | 30 | 0.87 | 2266 < 0.001 |
| 0 W | 270 | 33 | 0.85 | 2151 < 0.001 |
| 0 W | 270 | 117 | 0.13 | 47 < 0.001 |
| 25 WNW | 302 | 45 | 0.74 | 1641 < 0.001 |
| 25 WNW | 299 | 81 | 0.37 | 414 < 0.001 |
| 25 WNW | 296 | 90 | 0.29 | 248 < 0.001 |
| 15 WSW | 255 | 52 | 0.66 | 1320 < 0.001 |
| 15 WSW | 246 | 2 | 1.00 | 2997 < 0.001 |
| 15 WSW | 241 | 87 | 0.32 | 306 < 0.001 |

**Table S2.** Eastern North American migratory monarch butterflies (*Danaus plexippus*) reared in environmental chambers simulating autumn conditions (day: 29°C, night 23°C and 77% RH) until pupation. Monarchs were reared on swamp milkweed (*Asclepias incarnata*) grown in commercial soil treated with either at 4, 8, 15 or 25 ng/g of clothianidin (neonicotinoid: CLO) insecticide or a control (0 ng/g of clothianidin) before being released with radio-telemetry tags and tracked using the Motus telemetry array (n = 29). The number of days and distance to first detection, as well as the direction (°) of flight after release in Guelph (2017) and Cambridge, ON (2018) were recorded. Each row represents an individual monarch butterfly.

## Telemetry tracking

| **CLO**  **Year No. days after** | | | **Distance** | **Cardinal** | **Direction** |
| --- | --- | --- | --- | --- | --- |
| **Treatment (ng/g)** |  | **release** | **(km)** | **direction** | **(°)** |
| 4 | 2017 | 4 | 4 | N | 47 |
| 4 | 2017 | 3 | 43 | ESE | 117 |
| 0 | 2017 | 3 | 14 | SE | 125 |
| 0 | 2017 | 3 | 14 | SE | 125 |
| 0 | 2017 | 3 | 14 | SE | 125 |
| 8 | 2017 | 3 | 14 | SE | 125 |
| 8 | 2017 | 3 | 14 | SE | 125 |
| 8 | 2017 | 5 | 63 | SSE | 149 |
| 0 | 2017 | 16 | 201 | SSW | 174 |
| 0 | 2018 | 3 | 52 | SE | 126 |
| 0 | 2018 | 8 | 64 | SSE | 147 |
| 25 | 2018 | 7 | 12 | SSE | 156 |
| 0 | 2018 | 1 | 12 | SSE | 156 |
| 0 | 2018 | 2 | 12 | SSE | 156 |
| 0 | 2018 | 1 | 12 | SSE | 156 |

| 0 | 2018 | 2 | 12 | SSE | 156 |
| --- | --- | --- | --- | --- | --- |
| 25 | 2018 | 2 | 12 | SSE | 156 |
| 25 | 2018 | 1 | 12 | SSE | 156 |
| 25 | 2018 | 1 | 12 | SSE | 156 |
| 15 | 2018 | 1 | 12 | SSE | 156 |
| 25 | 2018 | 2 | 12 | SSE | 156 |
| 15 | 2018 | 1 | 12 | SSE | 156 |
| 25 | 2018 | 7 | 12 | SSE | 156 |
| 25 | 2018 | 2 | 12 | SSE | 156 |
| 25 | 2018 | 2 | 12 | SSE | 156 |
| 15 | 2018 | 1 | 12 | SSE | 156 |
| 25 | 2018 | 8 | 162 | SSE | 161 |
| 25 | 2018 | 8 | 162 | SSE | 161 |
| 15 | 2018 | 8 | 95 | ESE | 165 |
